# Voluntary peer-led exam preparation course for international first year students: Tutees’ perceptions

**DOI:** 10.1186/s12909-015-0391-5

**Published:** 2015-06-18

**Authors:** Daniel Huhn, Wolfgang Eckart, Kianush Karimian-Jazi, Ali Amr, Wolfgang Herzog, Christoph Nikendei

**Affiliations:** 1 Department for General Internal and Psychosomatic Medicine, Centre for Psychosocial Medicine, University Hospital of Heidelberg, Thibautstraße 2, 69115 Heidelberg, Germany; 2 Institute for History and Ethics in Medicine, Ruprecht-Karls-University Heidelberg, Heidelberg, Germany; 3 Tutors of Heidelberg’s Tutorial for International Medical Students (HeiTiMed), Ruprecht-Karls-University Heidelberg, Heidelberg, Germany

**Keywords:** Evaluation, Exam preparation course, International medical students, Semi-structured interviews

## Abstract

**Background:**

While the number of international students has increased over the last decade, such students face diverse challenges due to language and cultural barriers. International medical students suffer from personal distress and a lack of support. Their performance is significantly lower than non-international peers in clinical examinations. We investigated whether international students benefit from a peer-led exam preparation course.

**Methods:**

An exam preparation course was designed, and relevant learning objectives were defined. Two evaluations were undertaken: Using a qualitative approach, tutees (*N* = 10) were asked for their thoughts and comments in a semi-structured interview at the end of the semester. From a quantitative perspective, all participants (*N* = 22) were asked to complete questionnaires at the end of each course session.

**Results:**

International students reported a range of significant benefits from the course as they prepared for upcoming exams. They benefited from technical and didactic, as well as social learning experiences. They also considered aspects of the tutorial’s framework helpful.

**Conclusion:**

Social and cognitive congruence seem to be the key factors to success within international medical students’ education. If tutors have a migration background, they can operate as authentic role models. Furthermore, because they are still students themselves, they can offer support using relevant and understandable language.

## Background

In light of demographic change as well as the ambition to stay competitive in a globalized knowledge society, international students have become an increasingly precious and sought-after immigrant group [1]. Worldwide, the number of students studying abroad has increased by 77 % over the last decade, reaching its peak with almost 3.7 million in 2009 [2]. Nearly half of these students are enrolled in only five countries: the US (18 %), the UK (10 %), Australia (7 %), Germany (7 %) and France (7 %). Within the European Union, the UK accounts for the largest percentage of international students with 20.7 %, followed by Austria (19.4 %), Belgium (12.6 %), France (11.5 %) and Germany (10.5 %). As a result of this development, retention policies in EU countries have changed giving international graduates the opportunity to enter the labour market more easily after studying. The general trend in legal procedures, therefore, is one of liberalisation [1]. However, a diametrical development can also be observed in some EU countries that aim to reduce the allocation of student visas for non-EU residents [3].

International students face diverse challenges resulting from language and cultural barriers [4, 5], academic and financial difficulties [6], interpersonal problems [7], racial discrimination [8], lack of social support [9], alienation and homesickness [6, 8]. Focusing specifically on international medical students, it can be shown that they are exposed to greater disadvantages in the application process at universities [10–13], more often suffer from personal distress [14], have a reduced quality of life [15], and report insufficient support [12] and the loss of social contacts [16]. These facts might be responsible for international medical students’ higher drop-out rates [16, 17], prolonged duration of study [18, 19] as well as poorer results in written, oral and clinical examinations [19–26].

In medical education, peer-assisted learning (PAL) has become an established teaching method. It is not only effective, but comparable to faculty staff-led training in diverse areas of medical education [27–34]. Peer programmes are also popular and valuable in promoting international students’ interactions with their domestic counterparts [35], thus addressing some of the challenges noted. Peer-led tutorials can contribute to two aspects in this context: subject matter previously taught in lectures and seminars can be reviewed and discussed in more detail [4], and the sessions provide an informal atmosphere, enabling international students to share their concerns with peers and promoting the establishment of a peer community [36]. Furthermore, tutors’ and tutees’ cognitive and social congruence seems to have positive effects [37, 38]. The relaxed learning atmosphere makes it easier for students to talk about their difficulties and ask questions [39]. It has been documented that individuals working with international students should be culturally sensitive and appropriate [40]. To our knowledge, no evaluations of exam-centred, peer programmes for international students in undergraduate medical education have been conducted. The purpose of this study was to undertake such evaluation using both quantitative and qualitative approaches. Our tutorial programme seems to be innovative in its combination of social and cultural aspects as well as facets related to academic achievement.

## Methods

### Aim of the study

The study aimed to determine whether a voluntary, peer-led, exam preparation course for first year international medical students is a feasible and acceptable method for preparing students for tests and exams, and to investigate tutees’ attitudes towards such a course. Tutees were asked to rate the course’s atmosphere and content, the tutors’ performance, their personal progress and assessment of the course by means of written evaluations of each session. At the end of the semester, a selection of tutees participated in semi-structured interviews with respect to their attitudes, apprehensions, feedback and suggestions. Results were analysed to assess the course’s feasibility and benefits, and to identify crucial aspects for successful teaching and tutor training.

### Definition of learning objectives

In Heidelberg, tutorials for international medical students have a long history and are based on an initiative by the Institute of Ethics and History in Medicine. To assess international students’ expectations and needs, we conducted focus group interviews with 10 first-year international students in the 2013/2014 winter term. We also interviewed other German medical faculties regarding existing programs for international students [41] and conducted a literature review of this topic. Based on this information, learning objectives for the peer-led exam preparation course were defined, following principles of curriculum development by Kern [42]. The course aimed to equip international first-year students with two skills: 1) essential knowledge and skills beneficial for tests and exams during the first semester; and 2) a degree of familiarity with living and studying in a German city.

Learning objectives were defined for each meeting as follows: By the end of each session tutees will have achieved cognitive proficiency in the thematic area being taught and will also have improved their skills in medical terminology. Furthermore, tutees will have gained confidence in their abilities, as well as information about the exams and related requirements. They will have had the opportunity to ask questions at any point. Ultimately, by the end of each session tutees will have experienced a friendly environment, offering them social support.

### Design of the voluntary exam preparation course

The course was intended to be supportive, preparing international students for tests and exams during the first semester. It was offered on a regular basis to address all subject matter. In the first semester, 17 sessions took place in total, i.e. on a weekly basis for the 15 semester weeks with two additional dates included to meet the students’ demands. The first session was different from the others. It was designed as a launch event, in which international students could get to know each other, as well as find out more about the course’s format and tutors. Participants received general information about studying, living and working in Heidelberg. The following sessions lasted for at least 2.5 h each and dealt with relevant topics in anatomy necessary for the next exam.

Tutors displayed self-designed PowerPoint presentations, focusing on the most recently taught medical subject matter. With the help of these presentations, tutees were expected to review lecture content and ask questions about facts they did not understand. It was intended for tutees to start discussions with each other about the given topics, with tutors facilitating the process and offering support. Tutees were asked to complete fill-in-the-blank-texts and to write short tests giving them the opportunity to assess their performance level. Beside syllabus related work, the tutorials aimed to provide students with the opportunity to ask questions about university topics as well as addressing private challenges and problems. This was thought to be especially important in the first weeks of study, when international students faced the most challenges in adapting to their new setting.

### Peer tutors and tutor training

The exam preparation course was independently led by two peer tutors. They were recruited on a voluntary basis from the sample of international medical students in senior years. The most important criteria for their employment were their willingness and motivation to work with international first-year students. Both tutors were in their fifth-year and had a migration background. One is a citizen of Jordan who first came to Germany to study medicine. The other was born in Iran but had moved to and grown up in Germany. The tutors were thus well aware of the issues and experiences faced when starting studies in a foreign setting. To enable the first-semester students to benefit from their more advanced fellow students’ personal clinical experience, a traditional peer teaching system was preferred over the implementation of a reciprocal approach. The tutors were provided with financial compensation from the medical faculty by receiving contracts as student assistants.

Tutors were trained in two 2-h sessions. The training was led by a consultant of internal medicine and a psychologist, thus ensuring appropriate technical and didactic training. Each topic was reviewed. Questions were discussed and answered. Tutors were provided with teaching materials, e.g. a detailed presentation to give them a better idea of how to structure tutorials. Tutors were encouraged to be aware of participants’ needs, and were free to select topics based on the forthcoming exams or other issues that arose. In addition to this theoretical input, tutors were given an introduction to culturally sensitive behaviour [40] and the provision of structured and motivating feedback [43].

### Tutees

International students at the medical faculty of the University of Heidelberg were informed about the course in the first weeks of their studies and were free to enrol. A detailed description of the participating tutees will be presented in the results section.

### Qualitative evaluation of the course by tutees

Tutees were asked for their thoughts and comments on the course in semi-structured interviews at the end of the semester. Participating in such an interview was on a voluntary basis. Volunteers were provided with book vouchers amounting to 10€ each. Interview questions were developed based on an in-depth literature review as well as discussion among a team of experts. The interview guideline was constructed in a semi-structured format [44–46], containing mainly open-ended questions, followed by encouraging and clarifying questions if required. The interviews lasted between 10 to 30 min each and were conducted in a seminar room at the university hospital. During the interviews participants were asked about (1) reasons for participating in the exam preparation course; (2) what they had learnt in the course, and what was good or bad about this; (3) how they had developed knowledge, and what was helpful or difficult in this regard, and (4) suggestions to improve the course in future (see the interview guideline in the Appendix). The individual face-to-face interviews were conducted by one trained interviewer (DH), following the semi-structured interview guideline, and audio recordings were made.

### Quantitative evaluation of the course by tutees

In a parallel approach, all course participants were supplied with 14-item evaluation questionnaires after every course session. We wanted to get participants’ feedback as well as specific information about every session. The evaluation aimed to measure different levels of contentment as well as confidence. Satisfaction with the session’s content and atmosphere, support by tutors as well as fellow students and the perceived individual learning effect were assessed using a positively worded 6-point Likert scale (1 = I fully agree; 6 = I strongly disagree). Regarding newly gained confidence, the following aspects were rated using the same scale: feeling better prepared for the coming tests, feeling less anxiety about future tests and being sure to benefit from things learnt in future semesters.

### Statistical analysis of questionnaires and qualitative analysis of semi-structured interviews

Descriptive quantitative data are presented as means ± standard deviation. For the qualitative data, audio files of the 10 interviews were transcribed verbatim and content analysis was undertaken based on principles of qualitative content analysis [47]. First, we conducted an open coding of all the interviews to search for recurring topics. Single or multiple sentences were identified as a code, representing the most elemental unit of meaning. Next, the codes were summarised into relevant themes for each participant, using the software MaxQDA (2010 version, VERBI GmbH, Berlin). As themes recurred across participants, they were then compared and adapted until a number of relevant themes for all participants could be defined. The assignment of respective codes to specific themes was conducted by two independent analysts. They subsequently discussed the coding, and if required, adjustments were made once consensus was reached.

### Ethics

The ultimate goal of the study was curriculum improvement. Consequently, the ethics committee of the University of Heidelberg waived the requirement for ethical approval for the study design described (Number: S-108/2014). The study was conducted in accordance with the declaration of Helsinki (revised form, Fortaleza (Brazil), 2013) [48]. All participants gave written informed consent and study participation was voluntary.

## Results

### Student sample

Twenty-two (9 male, 13 female) students participated in the voluntary exam preparation course, comprising 17 sessions between October 2013 and February 2014. All participants were first year international students. Up to 10 % of approximately 360 students, who start studying human medicine in Heidelberg each year, report a migration background. Of these estimated 36 international students, 22 attended the course: resulting in a quota of 61 %. Table 1 shows detailed information about characteristics of this sample, as well as the group of students participating in the semi-structured interviews at the end of the semester.

**Table 1:** Characteristics of the HeiTiMed-participants as well as the students participating in the semi-structured interviews at the end of the semester

HeiTiMed-participants		
Number of tutees		22
Sex	Female	13
	Male	9
Mean age [years]		19.5 ± 1.7
Origin	Europe	7
	Middle East	6
	East Asia	4
	Central Asia	3
	Latin America	2
Group of students voluntarily participating in the interviews		
Number of tutees		10
Sex	Female	6
	Male	4
Mean age [years]		19.7 ± 1.3
Origin	European	3
	East Asia	3
	Middle East	2
	Latin America	2

### Main categories and themes resulting from qualitative analysis

Qualitative analysis of the transcripts revealed 126 relevant individual statements from tutees. From these statements, 20 themes were derived, which were consolidated into four relevant categories. The main categories included (A) reasons for participation in the exam preparation course, (B) technical and didactic learning experiences, (C) social learning experiences, and (D) conditions. Each of these main categories (A to D) contained 3–8 themes (i.e. A.1 to A.4).

### Definition of categories

In the following section, we provide definitions for the themes belonging to the main categories.

### Category A: Reasons for participation in the exam preparation course (24 quotations)

#### Theme A.1: Contact with other international students (8 quotations)

For international students, the contact with other international students seemed to be a pertinent motive to attend the course, because they ‘have the same difficulties with the language’ and they therefore ‘feel free there […] to speak’. Students could share the common experience of having ‘turned their lives upside down’, giving them a feeling of community.

#### Theme A.2: Senior student recommendation to attend the course (8 quotations)

Some students mentioned that senior students had told them that ‘without HeiTiMed [Heidelberg’s Tutorials for International Medical Students] [they] would not have passed the tests’ in the first year, indicating that this course ‘help[ed] foreign students very much’ to meet the high performance requirements. These findings suggest that senior students’ recommendations provided some direction for the international students at a time of uncertainty.

#### Theme A.3: Heavy workload at the start of university education (5 quotations)

International students perceived a heavy workload at the start of their university careers. One student stated he was told in the first week ‘to know 250 pages in 8 days’, which resulted in him ‘want[ing] to flunk out’ the next day. The first days at university felt overpowering and stressful to many international students, giving them the feeling of ‘never make[ing] it anyway’, leading to the question ‘what [were they] doing here’? They urgently needed assistance.

#### Theme A.4: Conscious decision to seek assistance (3 quotations)

Some of the international students had seen the advertisements for HeiTiMed and consciously decided to attend, because they thought ‘it [would] help [them] with anatomy and the syllabus’. They expected the international tutors to ‘explain everything to [them] at the beginning’ and ‘how [to] do it’. These findings indicate that international students not only attended the tutorial because of serious concerns, but also as a result of deliberate decisions.

### Category B: Technical and didactic learning experiences (52 quotations)

#### Theme B.1: Specification of content (11 quotations)

In the tutorial, international students gained insight into which topics were relevant for the exams and what literature was most appropriate to prepare for tests. Participants indicated that content was specified, and they were told not to ‘learn everything’ but to ‘learn this and that’, making learning easier for them. Tutors were found to ‘talk in a way that is easier to understand’ compared to lecturers’ technical jargon, which seemed to have facilitated their understanding.

#### Theme B.2: Senior students’ reports (11 quotations)

International students stated that the tutors, as senior students, were able to tell them ‘things and stories that they [had] experienced’. This information appeared to be valuable for the students, making the focus of the exams clearer, e.g., with hints like ‘foreigners always have problems with psychology and sociology’. Additionally, a first impression is that this helped make monotonous, theoretical learning tasks more accessible and understandable through practical examples. The fact that tutors were able to tell them ‘a bit about the clinic, not just what is in the books’ was experienced as helpful by the international students, and constituted a certainty of learning things ‘relevant for the future’, which they would ‘need to do later as doctors’.

#### Theme B.3: Provided teaching materials, like presentations, scripts or former exam sheets (8 quotations)

International students seem to have benefitted from being presented with teaching materials, provided by the tutors. It was noted that the tutors ‘repeated the most important images’ that would ‘come up in the exam’. Hence, there is evidence that students felt better prepared for upcoming tests and examinations.

#### Theme B.4: Clearly structured time schedule for learning (7 quotations)

Based on their own experiences, tutors were able to delineate a clear time schedule for studying. This was considered ‘very helpful’ by international students, because they knew they should ‘have learnt this and that’ at particular points. On the one hand, this appeared to ‘put [them under] time pressure’, but on the other hand, it seemed ‘like a stimulus for [them] to continue with learning’. There are signs that tutors’ experience allowed for a better estimation of how much time they should allow for preparation for respective tests.

#### Theme B.5: Content repetition and revision (7 quotations)

Students noted that when tutors ‘repeat things’ it felt ‘better for learning’ because you ‘can remember things better and not forget them so soon’. This continuous repetition of lesson content, in combination with explanations and the possibility to ‘ask questions there’, seems to have been seen as beneficial by the students.

#### Theme B.6: Delivery of mock exams (5 quotations)

That ‘before the exam there was always a test exam’ was considered helpful by the international students, giving them the possibility to experience a realistic self-assessment. Without this, the students would have had the feeling that already ‘everything was in [their] head’. It appears that the mock examinations showed them, in a gentle way, that ‘this is not true’, encouraging them to learn a little more.

#### Theme B.7: Active engagement with issues (3 quotations)

Some international students mentioned that the tutorials helped them to ‘also think, and […] not only learn a book page by heart’. Active engagement with the topics requires an understanding of them, and is a superior strategy to rote memorization. German students might just ‘memorize everything and pass the exams’, which would not be possible for international students because they ‘need[ed] to understand things, too’.

### Category C: Social learning experiences (37 quotations)

#### Theme C.1: Experiences with peers (26 quotations)

Students found it very reassuring that they were able to meet other international students who ‘have the same problems’ and, for example, ‘have to look up whole words, too’. They shared a view of themselves struggling in a new situation, confronted with both demanding study and the feeling of being alone in a foreign country. They were able to establish friendly relationships with each other, where nobody ‘fears being mocked because of errors’, which contributed to their well-being. Finally, there were initial indications that the social interaction within the framework of the tutorial enabled students to have more lucid learning experiences, as ‘all people explained something’ and it was possible to ‘see if you know a lot compared to the others’.

#### Theme C.2: Experiences with tutors (11 quotations)

One impression is that the international students viewed their tutors as being familiar with the students’ situation because of their own ethnic backgrounds. Therefore, the tutors seemed to ‘know how [they] feel’ and also to have had ‘the same sort of anxieties’, which gave them the opportunity to serve as role models for the new students. Tutors appeared to be able to offer better support based on their own experience, e.g., making comments like ‘Don’t be afraid, everything will work out in the end’.

### Category D: Frame conditions (13 quotations)

#### Theme D.1: Structure of tutorial sessions (5 quotations)

The tutorial course was structured more like a seminar than a lecture. Some of the students pointed out that the ‘classroom atmosphere [like] at school’ made interactions more personal. The smaller group size may have made it easier to relate to one another, so that it was possible to ‘ask questions’ and tutors could ‘take their time and move forward more slowly and explain everything’.

#### Theme D.2: Anxiety-free atmosphere (5 quotations)

International students experienced the tutorial as a place where they could be themselves, and were able to ‘ask questions without […] feeling awkward’. It was considered that ‘make[ing] mistakes was possible there’, without being ‘laughed at’. These facts seem to have contributed to the experience of the tutorial as an anxiety-free space.

#### Theme D.3: Tutors’ personal characteristics (5 quotations)

Tutors’ personal characteristics may have played a major role in making the tutorials beneficial. One student pointed out that the tutors are not ‘just people doing this in their spare time’ but rather ‘are committed’. They were perceived to have done an excellent job, being able to ‘explain these things in a way that [students] could understand them’.

### Tutees’ acceptance of the voluntary exam preparation course

A total of 319 evaluation questionnaires were completed over the 17 sessions by 20 students, corresponding to a response rate of 85.3 %. Results for these evaluations are shown in Table 2.

**Table 2:** Participants of the exam preparation course were provided with evaluation questionnaires, consisting of 14 items, using a 6-point Likert scale (1 = I fully agree to 6 = I fully disagree; for the last item: 1 = excellent to 6 = inadequate) after every course session. Across all course sessions and participants, 319 questionnaires were completed, with a response rate of 85.3 %

Items	M	SD	Range	N
I liked the atmosphere of today’s session.	1.2	.52	1 – 4	319
The learning objectives were clearly defined.	1.2	.52	1 – 5	319
I could follow the tutorial’s content well.	1.24	.61	1 – 4	319
I was able to ask questions about all things I did not understand.	1.2	.51	1 – 4	319
I could benefit from the tutor’s support.	1.14	.42	1 – 4	319
I could benefit from fellow students’ support.	1.39	.76	1 – 5	319
I felt well integrated into the group.	1.25	.56	1 – 4	319
Repetition of medical contents was helpful to me.	1.14	.42	1 – 3	319
The PowerPoint presentation facilitated my understanding.	1.26	.58	1 – 6	319
I have developed new levels of understanding.	1.25	.56	1 – 4	319
I now feel better prepared for the next exams.	1.89	1.00	1 – 6	319
I am now less scared by the next exams.	2.64	1.48	1 – 6	319
I am sure that today’s content will be useful in my future studies.	1.3	.57	1 – 4	319
I give the session the following mark.	1.2	.45	1 – 3	319

## Discussion

The aim of this study was to determine whether an innovative course designed specifically for international medical students at the beginning of their studies is a feasible and acceptable method for preparing such students for tests and exams. We wanted to investigate the nature of tutor-to-peer and peer-to-peer mechanisms and other factors involved in this process. Qualitative interviews, conducted at the end of the semester, gave a detailed picture of the participants’ experiences with the course. All interviewees were highly satisfied with the course, indicating a range of benefits including technical and didactic benefits, as well as social learning experiences. The continuous quantitative evaluation via questionnaires complements this picture.

Regarding participants’ motives and influences underlying the decision to participate in the course at the very beginning of their studies, the study revealed that the anticipated contact with other international students was one of the most relevant factors. This can be explained by the well documented lack of social contacts of international students [16, 49]. International students participating in our study relied on and trusted older international students, who gave them a pertinent motive to attend the tutorials. Heavy workload at the beginning of the semester was another crucial point underlying students’ motives to join the course. Here, they were required to pass exams almost on arrival in their new home. Interviewed students hoped to experience relief from this double strain through the tutorials. International students experience more personal stress [50] and reduced health-related quality of life [15]. In this regard, it seems that these difficult psycho-social circumstances constituted a key reason for international students seeking extra support.

Regarding the technical and didactic learning experiences, students mainly described the benefits from the tutorial’s specification of content. It was useful for them to actively assess the most relevant exam topics and associated key literature. There is also some evidence that they benefitted from the tutors’ more informal use of language compared with the lecturers’ technical jargon. International students seem to face double language difficulties: they have to deal with an unknown, informal way of speaking in a given social situation; they also need to become proficient in the new technical terminology of medicine. The social and cognitive congruence between tutors and students [37, 39, 42, 51] seemed to have a great impact on participants: the similar social roles appear to enable the tutors to be empathetic with tutees’ concerns. The tutees, in turn, seem to benefit from this more informal approach. In a sensitive environment, they can approach the heavy, technical medical-content in an informal way. The students also benefit from the materials provided, e.g., PowerPoint presentations or past exam papers, as well as through the clear structuring of operations owing to the tutors’ experience. The international students benefit from the repetition of teaching content as well as from an active engagement with this content, which they view as superior to memorisation. These aspects provide evidence in support of the idea that repetition is an important precursor to learning processes [52], as well as to Craik and Lockhart’s [53] theory that deep processing promotes consolidation and inclusion in an associative knowledge network. Within this learning process, international students welcomed the opportunity to ask questions whenever they faced problems, another reference to existing social congruence [39]. Finally, the international students saw advantage in that the tutors were already in their senior years. They had experience to share, as well as a highly practical view on the relevance of specific content. This last fact confirmed our assumption, that a non-reciprocal teaching approach [54, 55] must be used in this context, as international students cannot stabilize themselves during this vulnerable period and need tutors to function as role models [56, 57].

Regarding social learning experiences, interpersonal exchanges with like-minded and friendly people seem to be the most relevant issues among the interviewed international students. The contact with other international students was considered valuable, promoting an understanding that they are not the only ones experiencing problems with language, learning or adaptation to a new system. The course atmosphere was perceived as trusting, where the fear of humiliation was low and an open exchange among participants was possible. Another prominent issue was seen in the tutors having first-hand experience with the international students’ situation, further proof of the existing social congruence between tutors and students [39]. This result explains two perceived advantages: the tutors can offer much better and more applicable support owing to their own experience [37], and these tutors can serve as excellent role models, showing students what can be done in their specific situation [57].

With respect to framework conditions, the study revealed that the course’s seminar-like nature was important. Students appreciated the personal atmosphere of the small group, comparable to the situation in an actual lesson. Moreover, the establishment of personal relationships seems to be a key aspect, comparable to a social interface through which students are able to step out of anonymity and desperation. The course was experienced as a space where they could go without having to worry about anything. They could pose any question or make mistakes in what is sometimes described as a ‘fault-forgiving atmosphere’. The final point concerns the tutors’ personal characteristics. The tutorials were perceived to offer vital support to the students largely because of the tutors’ outstanding commitment and effective work. Tutors are seen as examples, counterparts and symbols of integration as well as teachers [58]. Based on these results, it becomes clear that the personal qualities of tutors must play a crucial role in light of their responsible task of guiding other students.

All the factors arising from our qualitative analysis seem to contribute to the quantitative evaluation results. These showed a very homogeneous picture: nearly all participants liked the course’s atmosphere, felt very well integrated into the group and very well supported by tutors and fellow students. They found the repetition of medical content to be very useful and developed new levels of understanding. The two questions addressing students’ concerns about their next examination and whether they felt better prepared for these, revealed ratings that were relatively weaker but still in the positive range. For the overall evaluation, they gave the course an excellent mark. It must be noted that despite attending a specific exam preparation course on a weekly basis, a feeling of insecurity still remained among students, indicating that the phenomenon of exam nerves is an important issue in education.

### Limitations

The qualitative part of our study was limited by the number of participants owing to the methods used, which included interviews and analysis. Another limitation occurs due to interviews’ brevity that allows only preliminary statements. Participation in interviews at the end of the semester was voluntary, and this may possibly have led to biases in our analysis. Tutors were reimbursed for their work and volunteering students received book vouchers for their participation in the study. Both provisions could also have biased the results. Finally, although the analysis was performed according to principles of qualitative content analysis [47] and was verified by a second analyst, the evaluation might still be considered more subjective than a quantitative analysis. However, owing to its openness in terms of assessment technique and its use of detailed interviews and quotations, the analysis enabled us to draw a more complete picture of this topic and led to a number of new insights that a quantitative evaluation could not have yielded in isolation.

## Conclusion

To the best of our knowledge, the current study is the first to examine a voluntary, peer-led, exam preparation course for first year international medical students. The rationale underpinning the course development was that this specific group of students suffers from more distress, fewer social contacts and insufficient support. International students perform significantly more poorly in examinations compared with other students. Results demonstrate that social and cognitive congruence play an important role in tutorials for international medical students’ education. When tutors themselves have a migration background, they can operate as authentic role models; moreover, as they are still students themselves, the tutors facilitate learning through the use of understandable language and the establishment of a permissive learning atmosphere. These points suggest educational as well as social benefits in the employment of more international peers for international student tutorials. Further research should address the effectiveness of such programs, as well as the phenomenon of students’ exam nerves, using a multicentre approach.

## Appendix

### Individual interview guideline for international first year students

OPENING QUESTION:

- What were your reasons for attending HeiTiMed?

INTRODUCTORY QUESTION:

- What did you learn within the context of HeiTiMed? What was positive, what was negative?

KEY QUESTIONS:

- Which didactic approaches did you get to know in HeiTiMed? Which did you find especially helpful? What difficulties did you encounter?
- Was HeiTiMed helpful in regard to your studies (examinations, assurance etc.)?

ENDING QUESTION:

- Where do you see room for improvement in the HeiTiMed program? What should be changed?
